# Eight weeks of aerobic exercise, but not four, improves insulin sensitivity and cardiovascular performance in young women

**DOI:** 10.1038/s41598-025-86306-2

**Published:** 2025-01-15

**Authors:** Maha Sellami, Shamma Almuraikhy, Khaled Naja, Najeha Anwardeen, Hadaia Saleh Al-Amri, Mohammad Shoaib Prince, Amina Ali Aden, Alexander Doemling, Mohamed A. Elrayess

**Affiliations:** 1 College of Sport Sciences, Sport Coaching Department, Qatar University, P.O. Box 2713, Doha, Qatar; 2 Biomedical Research Center, Qatar University, P.O Box 2713, Doha, Qatar; 3 Drug Design, Groningen Research Institute of Pharmacy, Groningen University, Groningen, The Netherlands; 4 Wellness and Health Promotion, Department of Sport and Wellness, University of Doha for Science and Technology, Doha, Qatar; 5 Hamad Medical Corporation (HMC), P.O Box 3050, Doha, Qatar; 6 College of Medicine, QU Health, Qatar University, P.O Box 2713, Doha, Qatar

**Keywords:** Physical activity, Insulin sensitivity, Metabolomics, Bilirubin degradation products, Glutarate, Ribose, Biochemistry, Pre-diabetes

## Abstract

Regular aerobic exercise has a significant impact on glucose metabolism and lipid profiles, contributing to overall health improvement. However, evidence for optimal exercise duration to achieve these effects is limited. This study aims to explore the effects of 4 and 8 weeks of moderate-intensity aerobic exercise on glucose metabolism, lipid profiles, and associated metabolic changes in young female students with insulin resistance and varying body mass, seeking to determine the optimal duration for physiological adaptations. Twenty-eight physically semi-active female students were randomly assigned to 4-week (G4, n = 13, age = 23.31 ± 5.19, BMI = 24.78 ± 5.87) and 8-week (G8, n = 15, age = 21.8 ± 2.56, BMI = 24.95 ± 4.81) training groups. The aerobic intervention maintained an intensity of 40–70% of maximum heart rate (HRmax). 6-min-walk test (6MWT), handgrip strength tests, insulin, HOMA-IR, lipid profiles, and metabolic profiles were assessed pre- and post-intervention. Following the intervention, G8, but not G4, exhibited a significant decrease in HOMA-IR (-14.59%, p = 0.047). The improvement in HOMA-IR was accompanied by notable improvements in 6-MWT (+ 38.18%, p < 0.001) and handgrip strength (+ 11.62, p = 0.027 and + 17.59%, p = 0.013), and increased levels of bilirubin degradation products, ribose, and glutarate. The elevated levels of bilirubin degradation products, known for their antioxidant properties, suggested a potential antioxidative response triggered by prolonged aerobic exercise. Additionally, an increase in ribose and glutarate indicated improved metabolic flexibility and enhanced utilization of alternative energy substrates. The 8-week aerobic exercise regimen demonstrated enhanced insulin sensitivity, upper body strength, and cardiovascular performance in young females compared to a 4-week regimen by triggering specific metabolic adaptations. These findings emphasize the complex relationship between exercise duration, metabolic adaptations, and overall well-being in young women, providing valuable insights for optimizing exercise prescriptions in promoting metabolic health.

## Introduction

Physical activity (PA) is integral to a healthy lifestyle, offering numerous benefits^1^, with a multifaceted influence on glucose metabolism. One significant advantage of exercise is its ability to combat insulin resistance (IR)^2^, thereby reducing the risk for cardiovascular disease and type 2 diabetes (T2D)^3^. Regular PA, encompassing aerobic exercise and resistance training, enhances insulin sensitivity (IS) by improving glucose uptake during muscle contraction^4^. PA has both immediate and longer-term effects on IS. Immediate effects of PA include ameliorated insulin signaling and enhanced glucose uptake by cells, while longer-term effects involve improvements in body composition, reductions in adipose tissue inflammation and ectopic lipid deposition, enhancement of the metabolic capacity of the skeletal muscles, and boost in antioxidant status^5,6^.

Current evidence, from different studies, supports that PA of any bout duration is associated with positive improvements in health outcomes^7^. According to the American College of Sports Medicine (ACSM) and the Centers for Disease Control and Prevention (CDC), adults should engage in at least two days a week of activities that maintain or improve their muscular strength and endurance. The 2024 ACSM result survey suggested that aerobic exercise regimen improve metabolic function and reduce body fat by creating a caloric deficit and help in maintaining long-term weight loss^8^. Low and moderate exercise is beneficial for cardiovascular health; however, excessive strenuous exercise may negate some of these benefits, and potentially increase the risk of adverse cardiovascular events in certain individuals^9^. Nonetheless, the effectiveness of PA varies based on its type and duration; hence, periodization of training and selecting the optimal mode become crucial components in improving physical fitness and performance^10^. Conclusions drawn from the literature emphasize the long-term benefits of PA on the immune system, strength, and metabolic status, which become evident within eight to twelve weeks. Insulin sensitivity, in turn, is strongly related to the degree of PA^11^, as research has shown that the volume and intensity of an exercise regimen can have varying effects on whole-body insulin sensitivity, likely due to enhancements in both skeletal muscle and hepatic insulin sensitivity^12^. Moreover, the changes of insulin sensitivity can be used as a marker to personalize the amount of physical exercise that confers cardiovascular protection^11^.

The molecular mechanisms underlying the improvement of insulin sensitivity induced by exercise training are complex and not fully elucidated^11^. Metabolomics, a powerful tool in systems biology, plays a crucial role in understanding the impact of exercise on the body, and exploring the physical activity-health relationship^13^. This approach helps in understanding how exercise influences various metabolic pathways, energy metabolism, and biochemical processes, shedding light on the mechanisms underlying the health benefits of physical activity. We have previously identified, through retrospective studies, distinct metabolic profiles associated with fitness status in both insulin-sensitive and insulin-resistant non-obese individuals^14,15^. This study explores the effects of 4 and 8 weeks of aerobic training in young, semi-active, or sedentary female students with IR and varying body weights, aiming to determine the optimal duration for physiological adaptations, particularly in glucose metabolism markers, then uncover the metabolic profiles associated with improvements of the training regimen.

## Methods

### Population

Twenty-eight physically semi-active female students (aged 20–30) from Qatar University voluntarily participated. Informed written consent was obtained from each participant. inclusion criteria included a BMI within 20–30 kg/m^2^ and absence of chronic diseases. Physically active status was defined as walking for more than 150 min, two to three days per week, measured using an IPAQ-modified version of the standard PA questionnaire. Using simple randomization ensuring equal chances^16^, participants were assigned to two groups: 4 weeks training (G4, n = 13) and 8 weeks training (G8, n = 15). This study aligns with the World Medical Association Declaration of Helsinki—Ethical Principles for Medical Research Involving Human Subjects. All protocols were approved by Qatar University (QU-IRB 1798-EA/23) and has received Expedited Review according to Qatar Ministry of Public Health (MoPH) regulations.

### Training session

The training program, adhering to American College of Sports Medicine (ACSM) and American Heart association (AHA) recommendations^17–20^ comprised aerobic exercises with progressive intensity (40–60% of HRmax and 50% of VO_2_ max initially, progressing to 60–70% by the 4th or 8th week). The maximum heart rate (HRmax) was estimated using the age-prediction formula: 220–20. VO_2_ max was not measured directly, but estimated using the formula of Burr et al.^21^ . VO_2_ max estimation was only used to follow up with training intensity progression and adaptation. Both groups trained three days/week for 50 min per session. The Metabolic Equivalent of Task (MET) values were adjusted based on IPAQ responses to quantify daily activities. MET was utilized for intensity and energy expenditure, expressed similarly for individuals of different weights. Training sessions were conducted on an indoor court at the university’s sports facility and incorporated a combination of continuous aerobic exercises and interval training. The sessions featured a variety of activities, including walking, running, cycling, as well as flexibility and balance exercises. These activities were designed to enhance cardiovascular and pulmonary capacity, improve range of motion, and reduce the risk of injuries.

### Study measures

Anthropometric measurements, fasting blood samples, blood pressure, and heart rate were taken before and after intervention. Blood pressure (fully automatic wrist blood pressure monitor, Omron Healthcare, Japan) and heart rate (H10 polar cardio-frequencemeter, Polar, Finland) were measured before and after the training session to map out the dynamic interplay between exertion and recovery and to ensure a better control of their health status. Body composition (TANITA, Tanita Corporation, Japan) was measured before exercise (before and after training), early in the morning, after 8 h of fasting. The subject, barefoot and dressed in light clothing, was positioned on the scale, and the measurements for weight (kg), body fat percentage (%), fat mass (kg), fat-free mass (kg), muscle mass (kg), bone mass (kg), basal metabolic rate (BMR, kJ), body mass index (BMI), degree of obesity, metabolic age, and height (cm) were recorded by the instrument.

Fasting blood samples were collected by a licensed nurse between 8:00 AM and 12:00 PM after a fasting period of 8 h. All samples were kept on ice, then centrifuged, and serum was separated within one hour after collection by centrifuging the blood for 15 min at 2500 rpm. For fasting blood glucose and lipid profile tests, the analyses were performed immediately using chemistry analyzer (Cobas 6000, Roche Diagnostics). The remaining serum was aliquoted and stored at -80°C. Insulin levels were measured in serum samples using Mercodia Insulin ELISA kit (UK) according to manufacturer’s instructions. Absorbance read using cytation5 (BioTek, imaging reader, USA). Homeostatic Model Assessment of insulin resistance (HOMA-IR) was calculated using the formula: HOMA = Fasting blood glucose (mmol/L) × Fasting insulin (mIU/mL)/22.5.

The samples for laboratory analysis were taken 24 h before the 6MWT and handgrip test to ensure that the biological markers or measurements being analyzed reflect a baseline state, not influenced by the transient psychological stress due to preparation for the effort and to not affect even minimally on the test performance^22^. A 6-min walking test (6MWT)^23–25^ and a handgrip test^26–28^ were performed before and after intervention. The 6MWT assesses endurance and cardiorespiratory capacity, conducted in a marked corridor, with baseline parameters measured before starting. The handgrip test measures grip strength using a dynamometer (CAMRY Digital Hand Dynamometer Grip), with three repetitions recorded, and a minimum 30-min rest between tests.

### Metabolomics and statistics

Established protocols were used for untargeted metabolomics of serum samples from all participants using Metabolon’s platform^29^. Metabolomics data for 1039 known identities and 259 unknown identities was batch-normalized, and imputed for missing values was carried out using the minimum value across batches from the median scaled data and log-transformed. From the subsequent statistical phases, unknown metabolites were disregarded. An examination of the before- and after-training groups was conducted using principal component analysis (PCA) to see whether any significant differences existed. An OPLS-DA model was used to determine which metabolites were the most discriminant between the intervention groups. Fold change and paired Students’ t test was employed for univariate analysis. Multiple testing correction was performed using the FDR method. Functional enrichment analysis was performed on all nominally significant metabolites list from the univariate analysis using Fisher’s exact test and was followed by the FDR multiple testing correction method. The sub-pathways were previously predefined using Metabolon, and those with less than three top hits were dropped. For laboratory parameters, normality was assessed using the Shapiro–Wilk test, outliers identified using the GOUT method, and statistical tests (parametric or non-parametric) performed accordingly. Baseline measurements were compared between groups using Student’s t/Mann Whitney U tests. ANCOVA was also conducted using a linear mixed-effects model with age as a covariate, group and activity as fixed effects, and a random intercept for each subject. The model was fitted using the lme function in R. We then used the emmeans package to obtain estimated marginal means (EMMs) and performed pairwise contrasts of activity levels within each group. R statistical computing language (version 4.2.1) and GraphPad Prism (version 10.0.3) were used for analyses. A p-value of < 0.05 was considered significant.

## Results

### Anthropometric measurements

Baseline characteristics and post-training measurements were compared between G8 and G4 groups (Table 1). The mean age of the participants was 21.8 ± 2.56 years for G8 and 23.31 ± 5.19 years for G4, with no significant difference between the groups. Both groups had an average height of 1.60 m. There were no significant changes in weight (G8: p = 0.768; G4: p = 0.309), BMI (G8: p = 0.785; G4: p = 0.999), body fat percentage (G8: p = 1; G4: p = 0.244), fat free mass (G8: p = 0.090; G4: p = 1), fat mass (G8: p = 0.486; G4: p = 0.585), and muscle mass (G8: p = 0.080; G4: p = 0.971) within the groups before and after the intervention. The effect size and 95% confidence interval (CI) for anthropometric measurements are presented in the supplementary table S1.

**Table 1:** Anthropometric measurements.

Parameter	G4 (n = 13)				G8 (n = 15)				Baseline comparison
	**Before (B1)**	**After (A1)**	**% Change**	**p**	**Before (B2)**	**After (A2)**	**% Change**	**p**	**p**
Age	23.31 ± 5.19		-	-	21.8 ± 2.56		-	-	0.33
Height (m)	1.60 ± 0.06		-	-	1.60 ± 0.07		-	-	0.99
Weight (kg)	63.49 ± 16.01	63.21 ± 15.71	-0.44	0.309	63.62 ± 12.52	63.76 ± 11.74	+ 0.22	0.768	0.980
BMI	24.78 ± 5.87	24.63 ± 5.75	-0.61	0.999	24.95 ± 4.81	24.91 ± 4.45	-0.16	0.785	0.934
Body fat %	0.31 ± 0.11	0.29 ± 0.11	− 6.45	0.244	0.31 ± 0.09	0.30 ± 0.08	-3.22	1.00	0.971
Fat free mass (kg)	42.95 ± 4.65	42.95 ± 3.94	0.00	1.00	43.17 ± 3.19	43.56 ± 3.22	+ 0.90	0.090	0.637
Fat mass (kg)	20.54 ± 11.67	20.34 ± 12.00	-0.97	0.585	20.52 ± 9.68	20.2 ± 8.99	-1.55	0.486	0.996
Muscle mass (kg)	40.77 ± 4.43	40.75 ± 3.75	-0.05	0.971	40.96 ± 3.03	41.35 ± 3.06	+ 1.04	0.080	0.890

Results of paired t-tests/Wilcoxon signed-rank tests for pre- and post-exercise comparisons within each duration group (G4 and G8). Percentage changes were calculated from group means to reflect the magnitude of changes. Data represents the mean ± SD for parametric and median (95%CI) for non-parametric.

### Performance measurements

Performance metrics for the G8 and G4 training groups are summarized in Table 2. The G8 group showed significant improvements in MET (p = 0.005), 6MWT (p < 0.001), handgrip (left) (p = 0.027), and handgrip (right) (p = 0.013). On the other hand, G4 results showed significant improvement only in 6MWT (p < 0.001) while no significant improvements were seen in MET(p = 0.305), handgrip (left) (p = 0.320) and handgrip (right) (p = 0.366). The effect size and 95% confidence interval (CI) for performance measurements are presented in the supplementary table S1.

**Table 2:** Performance measurements.

**Parameter**	**G4 (n = 13)**				**G8 (n = 15)**				**Baseline comparison**
	**Before (B1)**	**After (A1)**	**% Change**	**p**	**Before (B2)**	**After (A2)**	**% Change**	**p**	**p**
Metabolic equivalent (MET)	975 (622–1480)	1233 (958–1986)	+ 26.46	0.305	1084 (816.5–2165)	2013 (1251–3133)	+ 85.70	**0.005**	0.299
6-min walk test (6MWT)	558 (489–570)	606 (585–696)	+ 8.60	** < 0.001**	567.27 ± 91.06	783.87 ± 161.12	+ 38.18	** < 0.001**	0.516
Handgrip (left)	21.83 ± 4.04	23.02 ± 5.73	+ 5.45	0.320	22.89 ± 5.16	25.55 ± 6.05	+ 11.62	**0.027**	0.554
Handgrip (right)	26.3 (20.3–26.5)	25.2 (19.5–27.6)	-4.18	0.366	24.62 ± 5.1	28.95 ± 6.87	+ 17.59	**0.013**	0.503

Results of paired t-tests/Wilcoxon signed-rank tests for pre- and post-exercise comparisons within each duration group (G4 and G8). Percentage changes were calculated from group means to reflect the magnitude of changes. Data represents the mean ± SD for parametric and median (95%CI) for non-parametric.

### Laboratory parameters

**Fig. 1:**
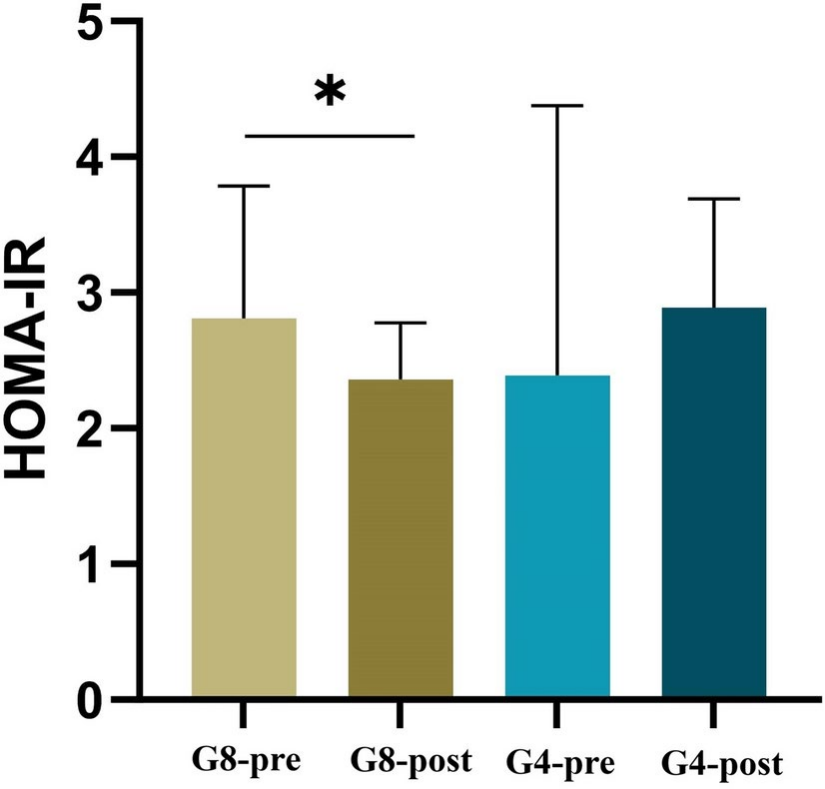
Bar plot showing the HOMA-IR level in the 8 weeks (pre and post) and 4 weeks (pre and post groups). Plots are based on median with IQR. * denotes p-value of < 0.05.

Laboratory parameters in G8 and G4 groups, before and after the intervention are summarized in Table 3. In G8, insulin levels decreased significantly (p = 0.044) along with a significant decrease in HOMA-IR (p = 0.047), while G4 showed no significant change (Fig. 1). An ANCOVA test revealed a non-significant improvement of HOMA-IR in G8 (p = 0.09) but not in G4. Supplementary table S2 reveals the results of ANCOVA test for all parameters. Fasting blood glucose (FBS) showed no significant change within the groups (G8: p = 0.790; G4: p = 0.368). Total cholesterol levels showed no significant change in either group (G8: p = 0.925; G4: p = 0.764). However, triglyceride levels significantly increased in G8 (p = 0.019), while G4 showed no significant difference (p = 0.484). No significant changes were noticed between HDL levels (G8: p = 0.513; G4: p = 0.169) and LDL levels (G8: p = 0.865; G4: p = 0.953). The effect size and 95% confidence interval (CI) for laboratory measurements are presented in the supplementary table S1.

**Table 3:** Laboratory parameters.

Parameter	G4 (n = 13)				G8 (n = 15)				Baseline comparison
	Before (B1)	After (A1)	**% Change**	p	Before (B2)	After (A2)	**% Change**	p	p
Insulin (mU/L)	13.03 ± 7.69	11.73 ± 4.92	-9.98	0.392	12.81 (9.05–16.62)	10.83 (8.23–14.27)	-15.46	**0.044**	0.810
FBS (mmol/L)	4.98 ± 0.44	5.11 ± 0.59	+ 2.61	0.368	4.97 ± 0.32	4.99 ± 0.2	+ 0.40	0.790	0.968
HOMA-IR	2.39 (1.76–3.94)	2.89 (1.73–3.53)	+ 20.92	1	2.81 (1.88–3.79)	2.4 (1.81–3.13)	-14.59	**0.047**	0.830
Total cholesterol (mg/dl)	182.46 ± 23.99	181.31 ± 25.43	-0.63	0.764	178 (157.5–203)	181 (162.5–205)	+ 1.69	0.925	0.794
Triglycerides (mg/dl)	64 (50–75)	57 (47–78)	-10.94	0.484	58 ± 13.78	70 ± 21.02	+ 20.69	**0.019**	0.677
HDL (mg/dl)	68.31 ± 13.81	65.85 ± 14.05	-3.60	0.169	62.89 ± 13.01	61.27 ± 11.57	-2.58	0.513	0.295
LDL (mg/dl)	101.46 ± 24.37	101.69 ± 24.61	+ 0.23	0.953	107.11 ± 33.79	107.8 ± 22.48	+ 0.64	0.865	0.621

Results of paired t-tests/Wilcoxon signed-rank tests for pre- and post-exercise comparisons within each duration group (G4 and G8). Percentage changes were calculated from group means to reflect the magnitude of changes. Data represents the mean ± SD for parametric and median (95%CI) for non-parametric.

## Metabolomics

### Multivariate analysis

**Fig. 2:**
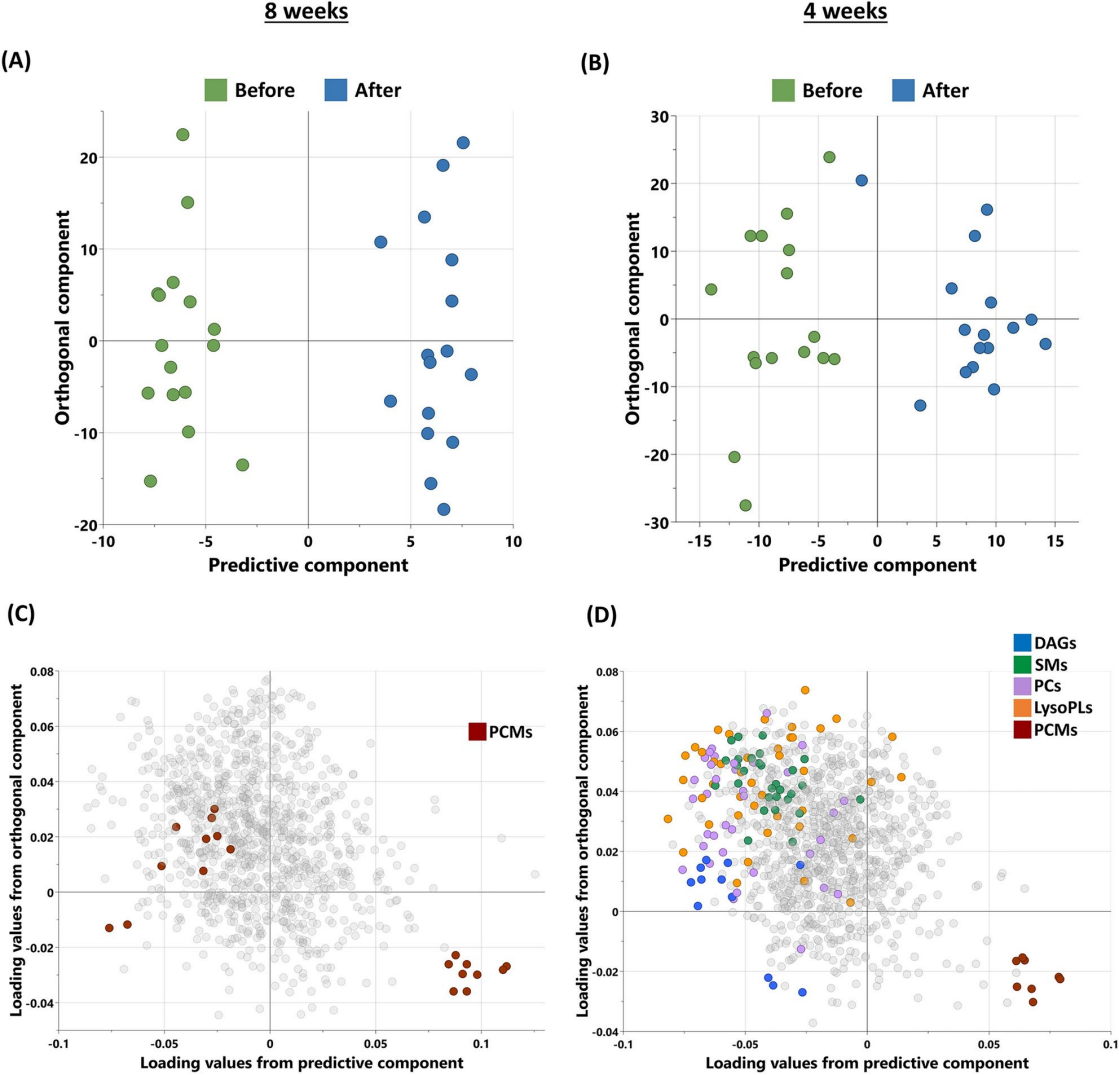
Metabolomics analysis of participants’ sera after 8 weeks of moderate physical training (n = 15) and 4 weeks training (n = 13). (A, B) OPLS-DA scores plot of the participants before and after physical training in G8 (R2Y – 0.966, Q2 – 0.214) and G4 (R2Y – 0.87, Q2 – 0.056) groups, respectively. (C, D) Loading plots depicting the metabolites from significantly enriched pathways of the same. [DAGs: Diacylglycerols, SMs: Sphingomyelins, PCs: Phosphatidylcholines, LysoPLs: Lysophospholipids, PCMs: Partially characterized molecules].

The metabolic signatures of the participants were analyzed using non-targeted metabolomics. OPLS-DA (Fig. 2) was utilized to identify the best distinguishing components in each group between pre- and post-intervention.

### Univariate analysis

**Fig. 3:**
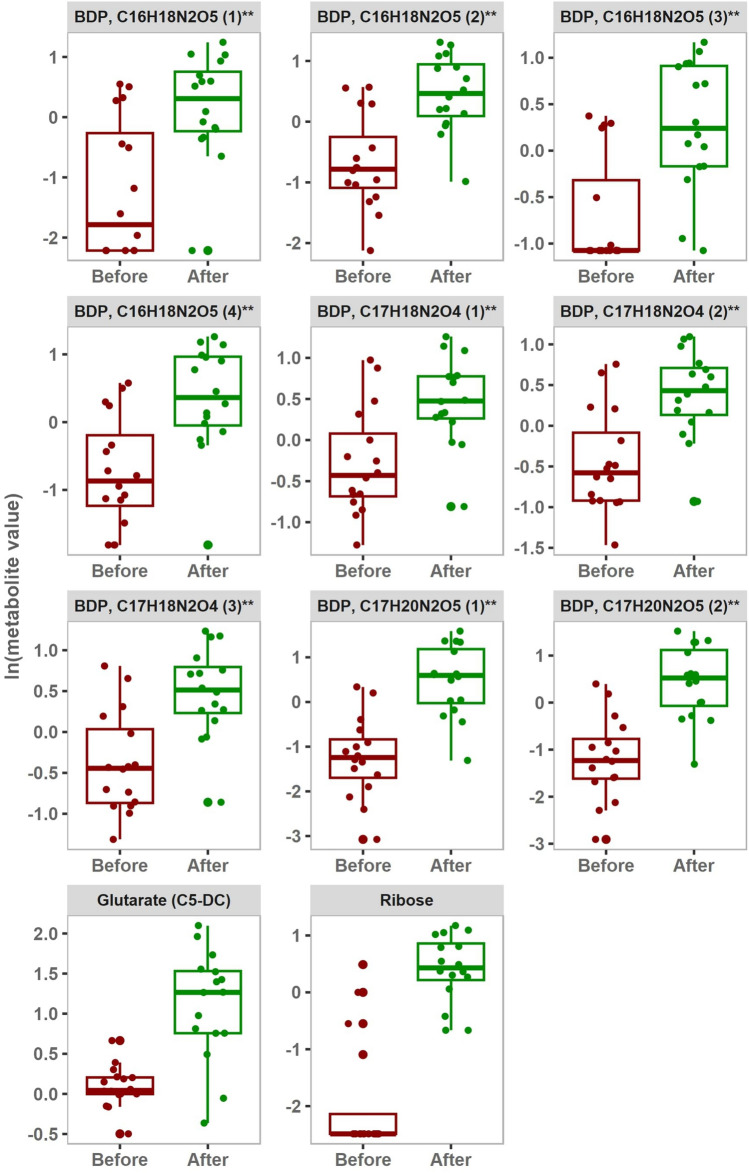
Metabolites differentiating between before and after intervention in 8-weeks training group. FDR < 0.05 are represented by box plots (paired student t-test). The y-axis represents the natural log values of metabolites. [BDP: Bilirubin degradation product].

**Fig. 4:**
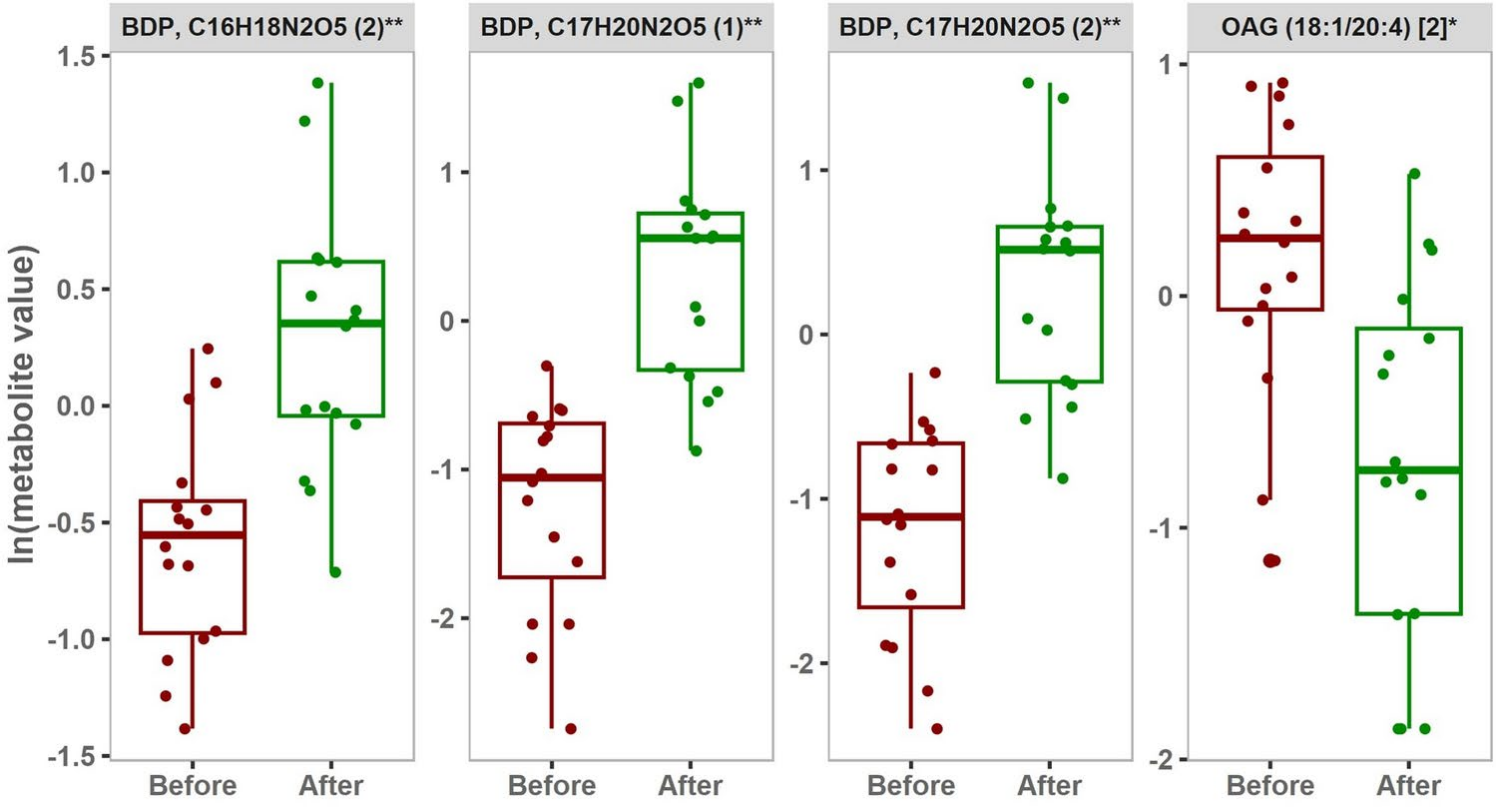
Metabolites differentiating between before and after intervention in 4-weeks training group. FDR < 0.05 are represented by box plots (paired student t-test.). The y-axis represents the natural log values of metabolites. [BDP: Bilirubin degradation product, OAG: oleoyl-arachidonoyl-glycerol].

Univariate analysis included paired Student’s t-test and fold change analysis to detect changes in metabolite levels between the before and after training groups. 12 and 13 metabolites were statistically significant after the intervention in G4 and G8 groups, respectively. However, after subsequent filtration based on fold change (FC) < 0.5 or > 2, G8 group had 11 metabolites and G4 group had 4 metabolites as statistically significant, and are represented in Figs. 3 and 4 respectively.

### Functional enrichment analysis

**Fig. 5:**
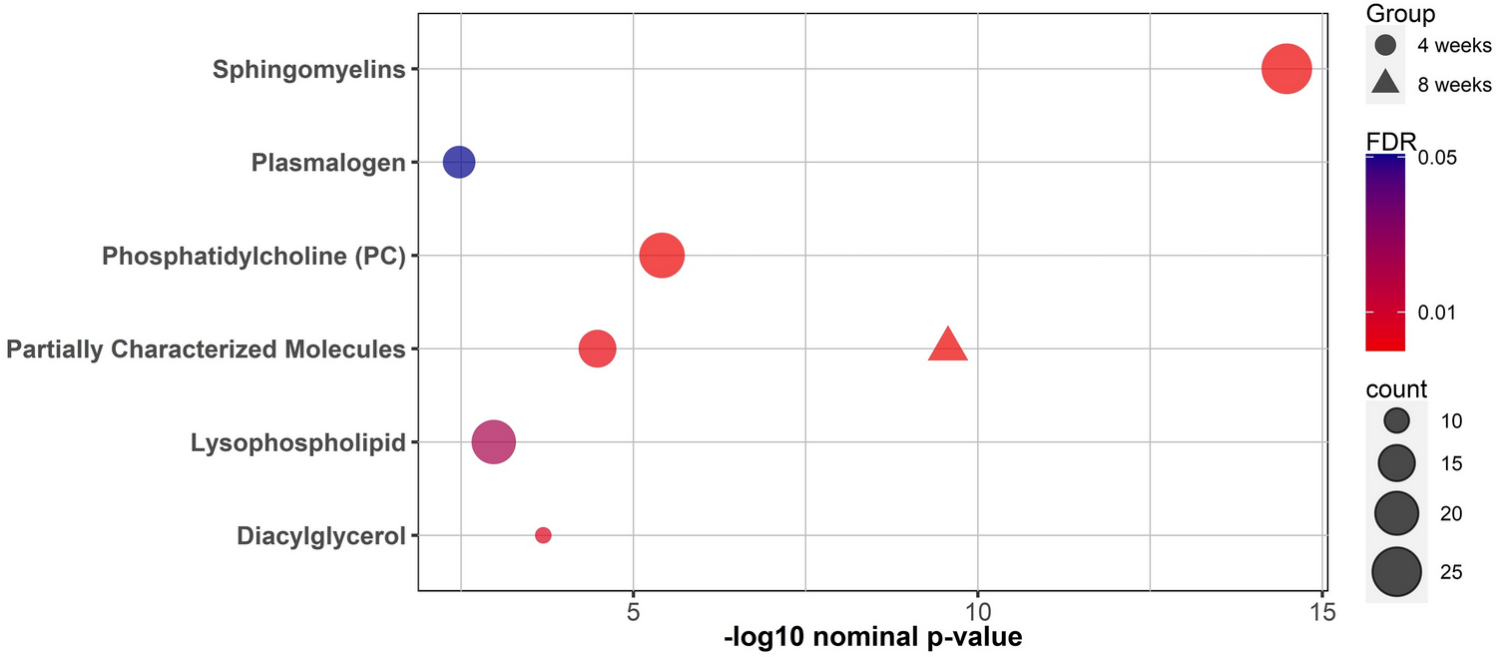
Bubble plot for the results of metabolite set enrichment analysis using Fisher’s exact test in both the training groups. The color of the bubble represents the weight of FDR significance (red; more significant, blue; less significant), size represents the number of significant metabolites from each sub-pathway and shape corresponds to the different training groups (circle: 4-weeks, triangle: 8-weeks).

Functional enrichment analysis was performed on nominally significant metabolites list from the univariate analysis using Fisher’s exact test and was followed by the FDR multiple testing correction method. The sub-pathways were previously predefined using Metabolon’s software, and those with less than three top hits were dropped. Results are presented in Fig. 5.

## Discussion

Physical exercise, including traditional and mind–body modalities, plays a crucial role in improving cardiometabolic health and physical fitness across diverse populations^30^. Both aerobic and resistance training have been shown to effectively reduce cardiovascular risk factors, enhance metabolic health, and improve overall fitness in individuals with and without metabolic dysregulation^31^. In this study, we investigated the impact of 4 and 8 weeks of aerobic training on young semi-active or sedentary female students with insulin resistance and lean to overweight status. The primary aim was to ascertain the optimal duration of exercise training required to induce physiological adaptations, particularly focusing on glucose metabolism markers, and to explore the metabolic pathways associated with each duration.

The aerobic training did not lead to discernible alterations in fat loss, muscle gain, or changes in body tissue distribution. Several factors may contribute to this lack of change, including the intensity and duration of the exercise regimen, dietary patterns, and the potential necessity for an extended training period to elicit measurable shifts in body composition. Both 4 weeks and 8 weeks of training demonstrated a notable increase in handgrip strength following aerobic training, with statistically significant improvements noted only in 8 weeks for the left hand, and the right hand. Recent research underscores the handgrip strength test as a prevalent method for assessing upper body strength in adults, serving as a valuable tool to identify populations at higher risk of cardiovascular issues such as heart disease and stroke^32^. On the other hand, a statistically significant increase in 6 min-walking distance was observed in both groups, with a more pronounced improvement over 8 weeks. The six-minute walk test is a commonly utilized assessment tool in diverse medical environments. It serves multiple purposes such as evaluating lung function in individuals with respiratory conditions, gauging exercise tolerance in those with cardiovascular diseases, and determining overall physical fitness across different demographic groups^33^. While aerobic exercise was shown to have a positive impact in increasing HDL and decreasing TC, LDL, and TG among women^34^, our data showed no statistically significant changes in HDL, LDL and TC. Unexpectedly, significant increase in triglycerides was detected in the G8 group following the intervention. This increase could be attributed to the uncontrolled diet, as no standard nutritional program was followed.

Our emerging data showed significant improvements in insulin and HOMA-IR in the G8 group, although no significant changes in body fat-free mass or mass were recorded. The enhancement of insulin sensitivity after 8 weeks is not surprising. Two longitudinal studies^35,36^ have yielded consistent results, demonstrating that regular physical activity, with lifestyle change, can reduce the incidence of diabetes by 58% in healthy individuals with insulin resistance (IR). Moreover, a systematic review and meta‐analysis of randomized controlled trials concluded that insulin, HOMA‐IR, and HbA1c were significantly lowered in exercise groups compared to control groups^37^. Our data suggests that four weeks may be insufficient to significantly impact insulin resistance, and relative benefit obtained from exercise will become more effective with prolonged exposure. These results emphasize the significance of advocating for increased frequency of exercise sessions for women with insulin resistance. Engaging in aerobic exercise can enhance glucose metabolism^38^; however, sustained improvements may require additional time for optimal regulation of glucose levels and glycogen reserves within muscle tissue. The enhancement of insulin sensitivity in 8-week regimen was concomitant with a significant increase in hand grip strength. This is in line with Li. et al.^39^ who reported that handgrip strength in adolescents was inversely associated with fasting insulin levels and HOMA-IR. This suggests that increasing muscular fitness may have beneficial effects for enhancing insulin sensitivity.

Metabolomics in exercise science serves as a powerful tool for studying how physical activity influences metabolism, offering valuable information for optimizing training programs, understanding physiological responses to exercise. Our metabolomics analysis revealed a rise in many bilirubin degradation products (BDPs) following 4 weeks and 8 weeks of exercise. Remarkably, the 8-week period showed an increase of a substantial number of these products, highlighting a progressive impact of exercise on bilirubin metabolism over time. Furthermore, our analysis revealed that the pathway for BDPs was significantly enriched only after 8 weeks.

Bilirubin, previously regarded as a waste product within the heme catabolic pathway, has emerged as a pivotal molecule with diverse functions. It not only exerts potent antioxidant effects, but also plays a crucial role as a metabolic hormone, highlighting its significance in various physiological processes^40^. BDPs are partially characterized molecules that arise from bilirubin breakdown, and could reflect its levels. Research on BDPs has recently gained attention. Fang et al. showed that BDPs were inversely proportional to the body max index^41^. Additionally, Saeed et al. reported that BDPs have protective anti-inflammatory properties, and are linked to a reduced risk of late-life cardiovascular disease events^42^. Moreover, Turi et al. demonstrated that these metabolites were associated with the protection from early-life wheeze and childhood asthma^43^. The rise of bilirubin levels after exercise is well documented. Aerobic exercise training can lead to elevated bilirubin concentrations through mechanisms such as increased heme catabolism and enhanced heme bioavailability^44^. Moreover, it has been shown that higher doses of exercise training led to greater increases in bilirubin levels^45^, and that elite athletes have significantly higher concentrations of serum bilirubin^46^. Given that bilirubin is a powerful antioxidant, its elevated levels could be, at least partially, attributed to the upregulation of antioxidant defense systems due to increased reactive oxygen species observed with exercise. Interestingly, the cross-talk between bilirubin and insulin resistance is firmly established, and bilirubin has been shown to increase insulin sensitivity by regulating various metabolic processes^47^. In fact, bilirubin’s antioxidant properties play a significant role in reducing oxidative stress, thereby potentially improving insulin sensitivity. Relatedly, recent findings suggest that elevated levels of bilirubin may have a significant protective effect against the development metabolic syndrome^48^. Our results are further corroborated by the significant increase in right and left handgrip in 8 weeks of exercise, which is in line with Kawamoto et al. who reported a strong association between an increased handgrip strength and serum bilirubin^49^. We hypothesize that the improvement of insulin sensitivity observed after 8 weeks of exercise could be, at least in part, attributed to the increase in bilirubin represented by its degradation products. Future studies are needed to focus on understanding the role and implications of bilirubin degradation products.

Our emerging data showed a significant increase in ribose following 8 weeks of exercise. To our best knowledge, this study is the first to document a raise of blood ribose levels after PA. Previous studies^50–53^ have focused on the effect of ribose supplementation on exercise performance. Indeed, ribose plays a crucial role in exercise by aiding in the recovery of ATP levels in muscle cells^51^. However, the ongoing debate surrounding the potential benefits or harms of ribose has endured for over fifty years, but definitive conclusion is still out of reach. While research suggests a potential link between increased ribose levels and some health issues like diabetes^54^, a study by Xue et al. showed that a nicotinamide and ribose combination substantially increased insulin sensitivity in healthy, middle-aged adults^55^. In fact, ribose is generated by the pentose phosphate pathway (PPP) the fundamental component of cellular metabolism. Interestingly, enhanced PPP was shown to countervail oxidative stress and reduce insulin resistance in diabetic rats^56^. Additionally, Lee et al. suggested that PPARδ, a regulator of glucose metabolism, may enhance insulin sensitivity by modulating glucose flux through PPP^57^. Importantly, Summermatter et al. showed that exercise, through the activation of PGC-1α, stimulates PPP in transgenic mice^58^. Moreover, the same authors suggested that the elevated PPP activity might be implicated in driving glucose uptake in skeletal muscles^59^. Our results suggest that the improvement in insulin sensitivity observed in G8 could be, at least in part, due to an increased PPP as indicated by the increase in ribose levels. Further research is needed to fully elucidate the role of ribose and PPP in the context of insulin resistance, and explore their potential implications for metabolic health.

The notable rise in glutarate levels following 8 weeks of exercise is undeniably intriguing. This observation suggests a potential link between this training regimen and metabolic pathways involving glutarate. Glutarate is an important intermediate of both tryptophan and lysine catabolism. Previous studies on the functions of glutarate has primarily focused on patients with glutaric aciduria type 1. However, very recently, a study^60^ reported that glutarate has an unexpected role in the regulation of metabolism. Glutarate was shown to act as competitive inhibitor of α-ketoglutarate-dependent dioxygenases, to modulate mitochondrial function by controlling pyruvate dehydrogenase complex via disruption of lipoylation, and to enhance the cytotoxicity of CD8 + T cells against target cells, consequently reducing tumor growth. In simpler terms, glutarate maintains oxygen consumption rates, acting as an alternative fuel source for mitochondrial oxidation, and promoting an increased glycolysis. Interestingly, in insulin-resistant conditions, there is a decrease in glycolysis, mitochondrial function, and oxygen consumption rates^61^. Nevertheless, further comprehensive studies are required to clarify the possible interplay between glutarate and insulin resistance.

While prior research has indicated that notable enhancements in insulin sensitivity after interventional studies are largely attributed to weight loss^2^, our study reveals that such improvements can be attained, at least partially, without a weight reduction, but rather through metabolic changes associated with exercise training. Moreover, most of these studies included hypocaloric diet, therefore it is difficult to evaluate whether physical activity alone has influenced the insulin sensitivity.

We acknowledge several limitations in our study that warrant consideration and future investigation. Primarily, the small sample size constrains the generalizability of our findings, necessitating validation in a larger, more diverse cohort to strengthen the robustness of our conclusions. Moreover, the lack of control over participants’ dietary habits and hydration status introduces potential confounding variables that may have influenced the observed outcomes. Additionally, the relatively young age of our cohort may have contributed to a more pronounced reduction in insulin resistance compared to what might be observed in older populations, highlighting the need for future research to explore the effects across diverse age groups. This age-related discrepancy underscores the importance of broadening the scope of our investigation to enhance the comprehensiveness and applicability of our findings. Furthermore, while our results are promising, they also illuminate the necessity for more in-depth exploration of the metabolic and molecular mechanisms underlying the exercise-induced improvements in insulin sensitivity.

## Conclusion

Eight weeks of aerobic exercise led to a decrease in insulin levels and HOMA-IR index, while also improving cardiovascular and upper body strength performance in young female students. This improvement in insulin sensitivity was associated with elevated levels of bilirubin degradation products, ribose, and glutarate. Our findings underscore the significant impact of aerobic exercise on metabolic health and physical performance in young women, highlighting the interconnectedness of exercise, metabolic processes, and overall well-being.

## Supplementary Information

Supplementary Information.

## Data Availability

Data are available from the corresponding author upon reasonable request.
