# Supplementary material for: Eight weeks of aerobic exercise, but not four, improves insulin sensitivity and cardiovascular performance in young women
Source: Sci Rep. 2025 Jan 15;15:1991. doi: 10.1038/s41598-025-86306-2 (PMC11735795; doi:10.1038/s41598-025-86306-2)
Supplement: Supplementary file 1 — Supplementary Information. [file 41598_2025_86306_MOESM1_ESM.docx]

**Supplementary table S1A**. The mean/median of differences and SD of differences, between before and after for 8-week groups for outcome variables are shown.

| **Parameter** | **Mean/median of differences (2-1)** | **SD** | **Cohen's d /Rank-Biserial Correlation (Effect Size)** | **95% CI** | |
| --- | --- | --- | --- | --- | --- |
| **Weight** | 0.133 | 1.722 | 0.077 | -0.821 | 1.087 |
| **BMI** | -0.047 | 0.650 | -0.072 | -0.407 | 0.313 |
| **Body Fat** | 0.000 | 0.020 | 0.000 | -0.010 | 0.010 |
| **Fat free mass** | 0.387 | 0.828 | 0.467 | -0.072 | 0.845 |
| **Fat mass** | -0.320 | 1.735 | -0.184 | -1.281 | 0.641 |
| **Muscle mass** | 0.387 | 0.793 | 0.488 | -0.052 | 0.826 |
|  |  |  |  |  |  |
| **MET** | 630 | NA | 0.780 | 429.000 | 1164.000 |
| **6WT** | 216.6 | 135.2 | 1.602 | 141.700 | 291.500 |
| **Handgrip (L)** | 2.66 | 4.174 | 0.637 | 0.348 | 4.972 |
| **Handgrip (R)** | 4.327 | 5.892 | 0.734 | 1.064 | 7.589 |
|  |  |  |  |  |  |
| **Insulin** | -2.082 | 2.859 | -0.728 | -4.003 | -0.161 |
| **FBS** | -0.014 | 0.263 | -0.054 | -0.166 | 0.137 |
| **HOMA-IR** | -0.547 | 0.895 | -0.611 | -1.087 | -0.006 |
| **Total cholesterol** | 0.507 | 20.600 | 0.025 | -10.900 | 11.910 |
| **Triglycerides** | 8.462 | 11.350 | 0.746 | 1.600 | 15.320 |
| **HDL** | -2.000 | NA | -0.210 | -6.000 | 3.000 |
| **LDL** | 0.687 | 20.020 | 0.034 | -10.400 | 11.780 |

Effect size is calculated using Cohen's d for parametric and Rank-biserial correlation for non-parametric variables. NA: Not applicable for non-parametric variables.

**Supplementary table S1B.** The mean/median of differences and SD of differences, between before and after for 4-week groups for outcome variables are shown.

| **Parameter** | **Mean/median of differences (2-1)** | **SD** | **Cohen's d /Rank-Biserial Correlation (Effect Size)** | **95% CI** | |
| --- | --- | --- | --- | --- | --- |
| **Weight** | -0.277 | 0.939 | -0.295 | -0.844 | 0.291 |
| **BMI** | -0.154 | 0.401 | -0.383 | -0.396 | 0.089 |
| **Body Fat** | -0.010 | 0.029 | -0.340 | -0.028 | 0.008 |
| **Fat free mass** | 0.000 | 1.576 | 0.000 | -0.953 | 0.953 |
| **Fat mass** | -0.200 | 1.285 | -0.156 | -0.977 | 0.577 |
| **Muscle mass** | -0.015 | 1.506 | -0.010 | -0.926 | 0.895 |
|  |  |  |  |  |  |
| **MET** | 283 | NA | 0.341 | -242.000 | 551.0. |
| **6WT** | 78 | NA | 1 | 39.000 | 153.000 |
| **Handgrip (L)** | 1.185 | 4.118 | 0.288 | -1.304 | 3.673 |
| **Handgrip (R)** | 1.2 | NA | 0.297 | -1.000 | 2.100 |
|  |  |  |  |  |  |
| **Insulin** | 0.005 | 2.502 | 0.002 | -1.585 | 1.595 |
| **FBS** | 0.123 | 0.475 | 0.259 | -0.164 | 0.410 |
| **HOMA-IR** | 0.03 | NA | 0.011 | -0.170 | 0.510 |
| **Total cholesterol** | -1.154 | 13.530 | -0.085 | -9.328 | 7.020 |
| **Triglycerides** | 6 | NA | 0.231 | -5.000 | 14.000 |
| **HDL** | -2.462 | 6.064 | -0.406 | -6.126 | 1.203 |
| **LDL** | 0.231 | 13.760 | 0.017 | -8.085 | 8.546 |

Effect size is calculated using Cohen's d for parametric and Rank-biserial correlation for non-parametric variables. NA: Not applicable for non-parametric variables.

**Supplementary table S2**. ANCOVA results for both groups.

| **Parameter** | **G4** | | |  | **G8** | | |
| --- | --- | --- | --- | --- | --- | --- | --- |
|  | **Estimate** | **SE** | **p-value** |  | **Estimate** | **SE** | **p-value** |
| **Weight** | -0.287 | 0.334 | 0.3962 |  | 0.156 | 0.334 | 0.6433 |
| **Body fat** | -0.0008 | 0.0058 | 0.1753 |  | 0 | 0.0058 | 1 |
| **Fat free mass** | 0.044 | 0.295 | 0.8832 |  | 0.3 | 0.295 | 0.3179 |
| **Fat mass** | -0.144 | 0.369 | 0.6995 |  | -0.206 | 0.369 | 0.5803 |
| **Muscle mass** | 0.025 | 0.282 | 0.9299 |  | 0.306 | 0.282 | 0.2857 |
| **MET** | 416 | 355 | 0.2496 |  | 1082 | 355 | 0.0047 |
| **6WT** | 106 | 27.1 | 0.0005 |  | 216 | 27.1 | <.0001 |
| **Handgrip (L)** | 1.54 | 0.998 | 0.1323 |  | 2.69 | 0.998 | 0.0115 |
| **Handgrip (R)** | 1.89 | 1.52 | 0.2243 |  | 4.01 | 1.52 | 0.0131 |
| **Insulin** | -1.21 | 1.23 | 0.3304 |  | -2.27 | 1.23 | 0.0747 |
| **FBS** | 0.05 | 0.09 | 0.6065 |  | -0.006 | 0.09 | 0.9486 |
| **HOMA-IR** | -0.296 | 0.316 | 0.3556 |  | -0.552 | 0.316 | 0.0907 |
| **Total Cholesterol** | -1.812 | 4.17 | 0.6668 |  | 0.475 | 4.17 | 0.91 |
| **Triglycerides** | 6.56 | 5.23 | 0.219 |  | 7.88 | 5.23 | 0.1424 |
| **HDL** | -1.19 | 1.84 | 0.5229 |  | -1.77 | 1.84 | 0.3434 |
| **LDL** | -2.125 | 4.2 | 0.6168 |  | 0.831 | 4.2 | 0.8445 |
